# Supplementary material for: MetaRibo-Seq measures translation in microbiomes
Source: Nat Commun. 2020 Jun 29;11:3268. doi: 10.1038/s41467-020-17081-z (PMC7324362; doi:10.1038/s41467-020-17081-z)
Supplement: Supplementary file 10 — Supplementary Data 7 [file 41467_2020_17081_MOESM10_ESM.zip › File2/Confidence_High_Taxonomy/88949_out.krona.html]

Javascript must be enabled to view this page.

members
magnitude
magnitudeUnassigned
count
unassigned
taxon
rank

88949\_out

2

2
2
superkingdom

1239
2
phylum

2
526524
class

order
2
526525

128827

SRS017521\_contig\_number\_10259
1
family
2

1505663
1
genus

species
1
1522

SRS146888\_contig\_number\_contig-100\_362.99483
